# HER2‐positive breast cancer brain metastasis: A new and exciting landscape

**DOI:** 10.1002/cnr2.1274

**Published:** 2020-09-03

**Authors:** Alexandra S. Zimmer, Amanda E. D. Van Swearingen, Carey K. Anders

**Affiliations:** ^1^ Women's Malignancies Branch National Cancer Institute Bethesda Maryland USA; ^2^ Duke Center for Brain and Spine Metastasis Duke Cancer Institute Durham North Carolina USA

**Keywords:** brain metastasis, CNS involvement, HER2‐positive breast cancer, T‐DM1, trastuzumab, tucatinib

## Abstract

**Background:**

Brain metastases (BrM) incidence is 25% to 50% in women with advanced human epidermal growth factor receptor 2 (HER2)‐positive breast cancer. Radiation and surgery are currently the main local treatment approaches for central nervous system (CNS) metastases. Systemic anti‐HER2 therapy following a diagnosis of BrM improves outcomes. Previous preclinical data has helped elucidate HER2 brain trophism, the blood‐brain/blood‐tumor barrier(s), and the brain tumor microenvironment, all of which can lead to development of novel therapeutic options.

**Recent findings:**

Several anti‐HER2 agents are currently available and reviewed here, some of which have recently shown promising effects in BrM patients, specifically. New strategies driven by and focusing on brain metastasis‐specific genomics, immunotherapy, and preventive strategies have shown promising results and are under development.

**Conclusions:**

The field of HER2+ breast cancer, particularly for BrM, continues to evolve as new therapeutic strategies show promising results in recent clinical trials. Increasing inclusion of patients with BrM in clinical studies, and a focus on assessing their outcomes both intracranially and extracranially, is changing the landscape for patients with HER2+ CNS metastases by demonstrating the ability of newer agents to improve outcomes.

## 1 INTRODUCTION

Breast cancer is the most common cancer in women, with 276, 480 new cases predicted for the year 2020 in the United States alone.^1^ It is also the second most common cause of brain metastases (BrM), with different reports indicating variable 10% to 30% incidence in breast cancer patients.^2^, ^3^, ^4^ The risk of BrM is subtype specific, with higher incidence among patients with human epidermal growth factor receptor 2 (HER2)‐positive and triple‐negative breast cancer.^5^, ^6^ In the HER2+ subtype of breast cancer, a diagnosis of BrM is common, affecting 25% to 50% of women with advanced disease.^7^, ^8^, ^9^, ^10^, ^11^ While the high incidence of BrM in the HER2‐subtype is likely multifactorial, it became more apparent after the arrival of trastuzumab, a HER2‐targeting monoclonal antibody (MAb) that improves survival and control of systemic disease but has lowcentral nervous system (CNS) penetrance, and is relatively ineffective at treating BrM.^12^, ^13^

### 1.1 Natural history

The incidence of BrM in breast cancer patients, before HER2‐subtype identification, was reported to be around 10% to 16% in symptomatic patients but 30% in autopsy reports.^4^, ^14^ The progressive improvement of systemic treatment of breast cancer led to increased survival as reports emerged of a higher incidence of BrM (28%‐48%) in stage IV patients treated with trastuzumab.^7^ Aiming to characterize CNS progression in patients with breast cancer in the clinical era of trastuzumab, a multicenter cohort of 1012 patients newly diagnosed with metastatic HER2+ breast cancer was followed in a prospective observational study from 2003 to 2006.^15^ Overall, 37% (377/1012) of patients developed CNS metastases, 7.5% (75/1012) at the initial diagnosis of metastatic disease, and 10.5% (106/1012) as the sole, initial site of progression. Trastuzumab was the main anti‐HER2 therapy available at the time, and only 5.5% of the patients had received it prior to study entry; however, 93% of patients received it during the follow‐up period, and before the first diagnosis of CNS metastases. The median time to development of CNS metastases was 10.8 months, and those patients had a shorter overall survival (OS) than those without CNS involvement (median 26.3 vs 44.6 months). The median survival after first diagnosis of CNS metastases for all patients was 13.0 months. A multifactorial analysis showed that systemic treatment with trastuzumab (HR 0.33; 95% CI: 0.25‐0.46; *P* < .001) or chemotherapy (HR 0.64; 95% CI: 0.48‐0.85; *P* = .002) decreased risk of death after CNS metastases, whereas CNS radiotherapy did not (*P* = .898). This is likely explained by the systemic control of disease.

The pivotal clinical trials that evaluated the adjuvant use of trastuzumab reported an overall low incidence of CNS as the first site of metastatic disease, with mixed results regarding a possible protective effect of trastuzumab.^16^, ^17^ An analysis of CNS relapses as the first event or at any time in the HERA trial data, which had a median follow‐up of 4 years, confirmed that the frequency of CNS relapses as the first recurrence event was similar between the group given 1 year of trastuzumab (2%, 37/1703 patients) and the observation group (2%, 32/1698 patients).^18^ Nevertheless, a subgroup analysis of 413 patients with available data regarding sites of progression after initial recurrence showed an increased incidence of CNS relapse in patients that did not receive trastuzumab compared to those who received trastuzumab (57%, 129/227 patients vs 47%, 88/186 patients; *P* = .06, respectively), again possibly related to improved systemic disease control, resulting in lower rates of CNS seeding.

A series of 123 patients with HER2+ breast cancer brain metastases (BCBrM) treated from 1998 to 2015, was subdivided into three cohorts based on the availability of new standard options of anti‐HER2 therapies: 1998 to 2007: trastuzumab; 2008 to 2012: lapatinib; and 2013 to 2015: pertuzumab and T‐DM1.^19^ While this is a small series with many limitations, as expected, median OS improved over time: 3.56 years for 1998 to 2007 (95% CI, 2.78‐6.05), 6.64 years for 2008 to 2012 (95% CI, 4.5‐8.58), and 7.55 years for 2013 to 2015 (95% CI, 4.37‐9.63) (*P* = .05). In a similar way, time to BrM diagnosis from initial breast cancer diagnosis increased over time, with a median time to brain recurrence of 2.63 years for 1998 to 2007 (95% CI, 1.34‐3.5), 2.61 years for 2008 to 2012 (95% CI, 2.11‐4.31), and 3.32 years for 2013 to 2015 (95% CI, 2.22‐6.01) (*P* = .05). Yet, the OS after the CNS metastases diagnosis was 1.51 years (95% CI, 1.24‐2.05) for the whole cohort and was not affected by the introduction of novel, systemic therapies (*P* = .24). However, those who received systemic anti‐HER2 therapy after the diagnosis of BrM had improved survival compared to those who did not (2.11 years [95% CI, 1.55‐2.60] vs 0.65 years [95% CI, 0.38‐1.25], *P* = .001).

Overall, the magnitude of benefit derived from anti‐HER2 systemic therapies in controlling systemic disease with improvements in OS cannot be understated. Moreover, receipt of anti‐HER2 therapy has also been associated with improved outcomes following a diagnosis of BrM. In addition, retrospective analyzes have consistently shown better outcomes for patients with HER2+ BrM, especially when also hormone receptor positive, in comparison to those with the triple negative subtype, likely due to the availability of effective, targeted therapies.^20^, ^21^, ^22^

## 2 PRECLINICAL STUDIES

### 2.1 Mouse models of HER2+ BCBrM

Several methods of modeling BCBrM in mice exist, each with its own benefits and caveats regarding the specific source of cancer cells (human vs mouse), tumor generation method (eg, direct intracranial implantation vs systemic inoculation), and analytical approach (eg, bioluminescence vs histology). As seen in Table 1, careful selection of the specific model must match the specific question(s) being tested, and resulting data must be appropriately interpreted based on the methods used. Some examples of important questions to consider include:

- Is an intact immune system required for this therapeutic intervention? If yes, then a syngeneic model with mouse cancer cells is needed.
- Is this gene/pathway of interest involved in the early metastatic process (eg, intravasation or colonization)? If yes, then direct intracranial implantation is ***not*** appropriate.
- Is the ability to monitor/detect individual BrM cells or micrometastases necessary? If yes, then bioluminescence is ***not*** appropriate.

**TABLE 1:** Comparison of some common methods for studying breast cancer brain metastases (BrM) in animal models

Method	Benefits	Caveats	Other considerations
Model			
Tumor source			
Human‐derived cell line	Human cancer cell biology Many established, well‐characterized lines Can easily differentiate tumor (human) vs environment (mouse) by sequencing Can genetically alter cells Can select for brain trophism	Immunocompromised mice Human cancer in mouse environment Cells change with prolonged culture	Lower incidence of extracranial disease in injected xenograft BrM models vs syngeneics
Mouse‐derived cell line	Immunocompetent mice Mouse cancer in mouse environment Numerous established, well‐characterized lines Can genetically alter cells Can select for brain trophism	Mouse cancer cell biology Cannot easily differentiate tumor vs environment (both mouse) by sequencing Cells change with prolonged culture	Syngeneic injected BrM models have shorter survivals vs xenograft models
Mouse‐derived spontaneous	Immunocompetent mice Mouse cancer in mouse environment Most faithful modeling of metastatic process	Mouse cancer cell biology Difficult to genetically alter the cancer cells Few models, currently Long latency to BrM Brain trophism harder to enrich Requires genetically engineered mice	
Tumor generation			
Direct intracranial injection	Established tumor biology Quick, high throughput Known, large tumor location Reproducible median survival PD/PK studies	Does not model early metastatic processes Specialized equipment, skillset needed Induces neuroinflammation Cancer‐naïve host	
Intracardiac injection	Reproducible median survival Minimally invasive surgery Models extravasation, brain colonization and outgrowth Average equipment and skillset needed	Variable BrM rate, especially with nonbrain‐trophic cells Systemic circulation of cells leads to extracranial mets Does not model intravasation Cancer‐naïve host	
Intracarotid injection	Reproducible median survival Minimal systemic circulation of injected cancer cells, fewer extracranial mets Models extravasation, brain colonization and outgrowth	Invasive, difficult surgery Specialized equipment, skillset needed Does not model intravasation Cancer‐naïve host	
Orthotopic injection	Closely models most of the metastatic process Noninvasive implantation	Variable BrM rate, especially with nonbrain‐trophic cells Long latency to BrM Frequently also develop extracranial tumors Surgery to resect primary tumor required	
Spontaneous	See above (tumor source)		
Analysis			
Bioluminescence	*In vivo* and *ex vivo* Noninvasive, real time Quick, high throughput Inexpensive Average equipment and skillset required	Moderate sensitivity Moderate spatial resolution No/difficult assessment of microenvironment	Cells must express luciferase Cells expressing reporters may activate immune response
MRI	*In vivo* Noninvasive, real time High‐spatial resolution Microenvironment assessment possible Can assess blood‐brain barrier permeability	Moderate sensitivity Moderate throughput Expensive Specialized equipment, skillset needed	
Two‐photon microscopy	*In vivo* Noninvasive, real time High sensitivity High‐spatial resolution Microenvironment assessment possible	Slow, low throughput Specialized equipment, skillset needed	Cells must express fluorescent proteins Cells expressing reporters may activate immune response
Histology	Highest sensitivity Highest spatial resolution Microenvironment assessment possible Average equipment and skillset required	*Ex vivo* only Terminal/invasive Moderate throughput Single timepoint per animal	Requires optimized histology methods and specific antibodies
FACS	Quick, high throughput Inexpensive Microenvironment assessment possible Average equipment and skillset required	*Ex vivo* only Terminal/invasive Moderate sensitivity No/limited spatial resolution Single timepoint per animal	Requires specific antibodies and/or cells expressing fluorescent proteins Cells expressing reporters may activate immune response

In the field of HER2+ breast cancer, the story of HER2 actually started in the brain and has been extensively characterized in animal models. The role of HER2 in cancer was first identified in a rat brain tumor model, and development of the HER2‐targeting MAb trastuzumab in animal models has provided a road map for future antibody‐based therapeutics (reviewed in Reference 23). Studies of HER2+ BCBrM have utilized the full arsenal of mouse models, ranging from direct implantation of human‐derived BC cells into the brains of nude mice to intravenous and intracardiac injection of cells to spontaneous metastasis models. More recently, the field has also been leveraging patient‐derived xenografts (PDXs) in immunocompromised mice. Early PDX models derived from tumorspheres of HER2+ primary core needle biopsies demonstrated a capacity for spontaneous metastasis to the brain as well as other organs.^24^ PDX models derived from HER2+ BCBrM have also been generated.^25^ However, the increasing importance of immunotherapies in the clinic has highlighted the need for more immunocompetent models. Syngeneic models of HER2+ BC that spontaneously metastasize to the brain, such as one recently characterized, are poised to become more standard in the field.^26^

### 2.2 Biology of HER2+ BCBrM

Extensive work in HER2+ animal models has provided potential explanations for why this subtype of breast cancer has a predilection for CNS recurrence and subsequent BrM. HER2, as an oncogene itself, may drive brain trophism, as HER2 induces a more mesenchymal state in breast cancer cells, increasing invasiveness and metastatic potential.^27^ Induced expression of HER2 in experimental models increases the size of BrM from intracardiac injections, and may alter the spatiotemporal growth of BrM within different brain regions toward favoring more posterior areas.^28^, ^29^

HER2's ability to increase BrM may be due in part to proposed interactions between HER2 and other receptors. Interactions between and signaling from HER2 and its family members, notably the epidermal growth factor receptor (EGFR) and HER3, have been implicated as driving factors in BCBrM (reviewed in Reference 30). BCBrM often overexpress HER2 and HER3, and can express mutated EGFR, even relative to primary tumors and other metastases.^31^, ^32^, ^33^, ^34^, ^35^ The brain microenvironment contains several HER family ligands, including neuregulins, which can cause dimerization and activation of these receptors in brain metastatic cells.^31^, ^36^ The HER2:HER3 association appears to be particularly important in BCBrM, as it may drive BrM through the release of matrix metalloproteases that can disrupt the blood‐brain barrier (BBB).^36^ Furthermore, the interaction between HER2 and HER3 is enhanced by Src activation and may be a mechanism of resistance to HER2‐targeting agents.^37^ Preclinical models have shown that HER2 can also heterodimerize with the neutrotropin receptor TrkB and be activated by the neutrotrophic factor BDNF, suggesting that paracrine signaling increases survival of HER2+ BCBrM.^38^ Interestingly, estrogen present in premenopausal women may further drive this BDNF/TrkB signaling, as well as the migratory and invasive capacity of breast cancer cells through paracrine signaling from ERα‐expressing astrocytes in the brain, further driving the growth of BrM.^39^, ^40^ Preclinical studies have also shown that HER2, along with EGFR, in BCBrM can alter proliferation by modulating DNA topoisomerase I through nucleolar localization of heparanase (HPSE).^41^

Additional features of HER2+ BC may contribute to its predilection for BrM. Truncated glioma‐associated oncogene homolog 1 (TGLI1) is highly expressed in HER2+ BC and has been shown to increase the incidence of BrM.^42^ TGLI1 may also contribute to radioresistance by increasing stemness and creating a “metastasis‐friendly” microenvironment through activation of astrocytes.^42^ Fatty acid binding protein 7 (FABP‐7) is a lipid binding protein found specifically in the brain. However, FABP‐7 is also expressed in BC cells, particularly in HER2+ cells and in BrM.^43^ FABP7 is thought to induce a more glycolytic, metastatic, and pro‐angiogenic state in BC cells, thereby enhancing the survival of HER2+ BC in the foreign brain microenvironment.^43^ Thus, beyond just HER2 expression and its interaction with other receptors, other aspects of HER2+ BC biology likely contribute to the brain metastatic potential of this subtype.

### 2.3 Blood‐brain and brain‐tumor barriers

The BBB is composed of tight junctions between various brain cells to prevent substances, including most cancer treatments, from crossing into the brain from circulation. However, this barrier is often altered in BrM, including changes in many of the cells that make up the barrier, leading to the concept of a substantially different blood‐tumor barrier (BTB).^44^ Seminal papers in different BCBrM mouse models, including HER2‐expressing models, demonstrated that the BTB is compromised in the vast majority of experimental BCBrM, enabling increased but heterogenous, and still often subtoxic, levels of drug uptake by BrM relative to normal brain tissue.^45^, ^46^, ^47^ Indeed, trastuzumab reaches preclinical brain tumors, but is not as effective at controlling intracranial disease in the clinical setting.^48^ Surprisingly, the distribution of trastuzumab does not appear to be dictated by vascular architecture, or lack thereof, in BrM.^47^ Small molecule HER2 inhibitors do not peform substantially better. Lapatinib does achieve higher levels in intracranial tumors of HER2+ BCBrM mouse models relative to normal brain, but the elevated levels in brain tumors are short‐lived (<12 hours), heterogenous due to differential permeability of the BTB, and below the concentrations reached in extracranial metastases.^49^ This limited exposure is due, in part, to active removal of lapatinib by P‐glycoprotein (Pgp) and breast cancer resistance protein (Bcrp) efflux pumps inherent to the BBB.^50^ Historically, efficacy of systemic treatments with most HER2‐targeting agents against BrM has been somewhat limited and inconsistent, though newer generation HER2‐targeting agents may circumvent these issues.^51^

Several approaches have been tested in HER2+ BCBrM mouse models to improve access of drugs through the BBB and BTB. Studies have demonstrated the feasibility of using MRI‐guided focused ultrasound in HER2+ BCBrM mouse models to disrupt the BBB and thereby increase trastuzumab delivery to BrM.^52^ Focused ultrasound has also been used with microbubbles to increase the accessibility of both chemotherapy and an antibody‐drug conjugate (ADC) in a HER2+ BCBrM mouse model.^53^ While these methods hold some promise, improved systemic agents that can better cross the BBB and BTB are needed.

### 2.4 Therapeutic development for HER2+ BCBrM

The development of targeted therapies for both treatment and prevention of BrM in preclinical HER2+ BCBrM animal models has been extensive, ranging from small molecule inhibitors, to antibody‐based strategies, to engineered cells under evaluation. Lapatinib, a small molecule inhibitor of EGFR and HER2, was the first HER2‐targeting agent characterized in a model of BCBrM.^54^ Animals treated with a higher dose of lapatinib (100 mg/kg) demonstrated significantly fewer large BrM after systemic inoculation of brain‐seeking BC cells relative to vehicle‐treated controls.^54^ Pazopanib demonstrated prevention potential against HER2+ BC brain micro‐ and macrometastases in preclinical models by reducing proliferation of tumor cells.^55^ Neratinib treatment completely prevented any BrM, large or small, in a spontaneous HER2+ BC mouse model with a high proclivity for spontaneous BrM.^26^ Newer HER2‐targeting agents, such as TAK‐285 and Epertinib (S‐222611), display improved efficacy over lapatinib, due in part to achieving higher concentrations in BrM.^51^, ^56^, ^57^

Beyond traditional small molecule inhibitors, additional novel treatment strategies have been characterized in HER2+ BCBrM models. As discussed above, antibodies targeting HER2, including trastuzumab, have been tested in these models, but with modest efficacy given inconsistent brain penetrance. However, improvements to the antibody‐based therapies, such as conjugation with other drugs or with peptides, have also been studied in HER2+ mouse models. An antibody conjugate of trastuzumab and melanotransferrin (BT2111) has been shown to reduce the number and size of BrM in a HER2+ mouse model.^58^ An anti‐HER2 antibody‐peptide conjugate, ANG4043, demonstrated significant efficacy in HER2+ BCBrM mouse models, likely due to its increased BBB permeability through receptor‐mediated transcytosis.^59^ A recent report from the Steeg laboratory demonstrated significant reduction of BrM outgrowth in 2 hematogenously generated HER2+ BCBrM models using a novel biparatopic HER2 antibody conjugated to tubulysin.^60^ This reduction in BrM occurred despite low, heterogeneous brain uptake of the antibody conjugate.^60^ Nanoparticle technology has also been explored as a method to increase BrM exposure to HER2‐targeting agents. Indeed, albumin nanoparticles demonstrated increased delivery of lapatinib to BrM in animal models, thereby inhibiting the growth of metastases and extending survival.^61^ Combination therapies have also been attempted with nanoparticle delivery, including a chemotherapy plus anti‐HER2 antibody combination, which reduced tumor volume in an intracranial HER2+ model.^62^

There is growing appreciation for the need of combination therapies in HER2+ BCBrM, as HER2‐directed therapy alone does not appear to always be sufficient. Activation of the PI3K/mTOR pathway in BCBrM, particularly in HER2+ disease, likely explains the need for combination strategies for HER2+ BCBrM (reviewed in Reference 63). Dual inhibition of PI3K and mTOR in HER2+ BCBrM PDX models improved survival in the absence of a HER2‐targeting agent, though some models with high‐genomic instability were resistant.^64^ Indeed, trastuzumab required addition of a brain penetrant PI3K/mTOR small molecule inhibitor (GNE‐317) or conjugation with a cytotoxic agent (TDM1) to improve survival.^48^ Combination strategies have expanded beyond the PI3K pathway. Partnering Src inhibitors with HER2‐targeting lapatinib induced cell cycle arrest and apoptosis, thus reducing the incidence and size of BrM in an intracardiac model.^37^ A triplet strategy combining trastuzumab, lapatinib, and an anti‐VEGFR2 antibody drastically improved survival in mouse models of HER2+ BCBrM by decreasing microvessel density in intracranial tumors, thereby increasing necrosis.^65^

Recent progress in the field of cell‐ and viral‐based therapeutics has also been applied to HER2+ BCBrM. Human‐derived neural stem cells secreting an anti‐HER2 antibody, when injected intracranially at the site of tumor implantation, increased survival of mice.^66^ Similarly, mesenchymal stromal cells (MSCs) infected with and secreting an engineered virus designed to target HER2 instead of its usual target decreased the incidence of BrM from systemically injected HER2+ BC cells with just one treatment.^67^ HER2‐CAR T cells delivered intraventricularly drastically reduced intracranial HER2+ BC tumors, including multifocal and leptomeningeal disease, thereby increasing survival.^68^ Engineered fibroblasts secreting trastruzumab caused tumor growth inhibition and increased survival in a HER2+ BCBrM mouse model when implanted distally (contralaterally) to the tumor.^69^ Even viruses alone have shown promise. An engineered adeno‐associated virus (AAV) vector, which causes neurons and astrocytes to create an antibody similar to trastuzumab, demonstrated significant efficacy with just a single intrathecal dose on both prevention and treatment of preclinical HER2+ BCBrM.^70^ Another adenovirus forcing generation of trastuzumab, when injected contralateral to an implanted intracranial tumor, demonstrated tumor growth inhibition and increased survival.^69^ Cell‐based and viral‐based strategies are rapidly being developed in the HER2 space and have shown significant promise for HER2+ BCBrM.

### 2.5 Human tissue‐based studies

Analyzes of patient specimens have further suggested an important role for HER2 in the biology of BrM. HER2 was identified as one of four “brain metastasis selected markers” for CTCs in patients with metastatic BC, where CTCs expressing these markers had increased propensity to spread to the brain.^71^ HER2+ BCBrM display increased HER2+ amplification and activation compared to primary tumors^32^, ^34^, ^35^ Indeed, one study identified HER2 (and RET) as being gained in patients' BrM relative to their primaries, and treatment of BrM subcutaneous PDX models with HER2 and RET inhibitors showed significant growth inhibition.^72^ BCBrM also demonstrate increased expression and/or mutation of HER2 family members HER3 and EGFR.^31^, ^33^ Thus, extensive preclinical and correlative work in the field of BCBrM has demonstrated the importance of HER2 and its family members in the biology and potential treatment of BCBrM.

## 3 CLINICAL MANAGEMENT OF HER2‐POSITIVE BrM

**FIGURE 1:**
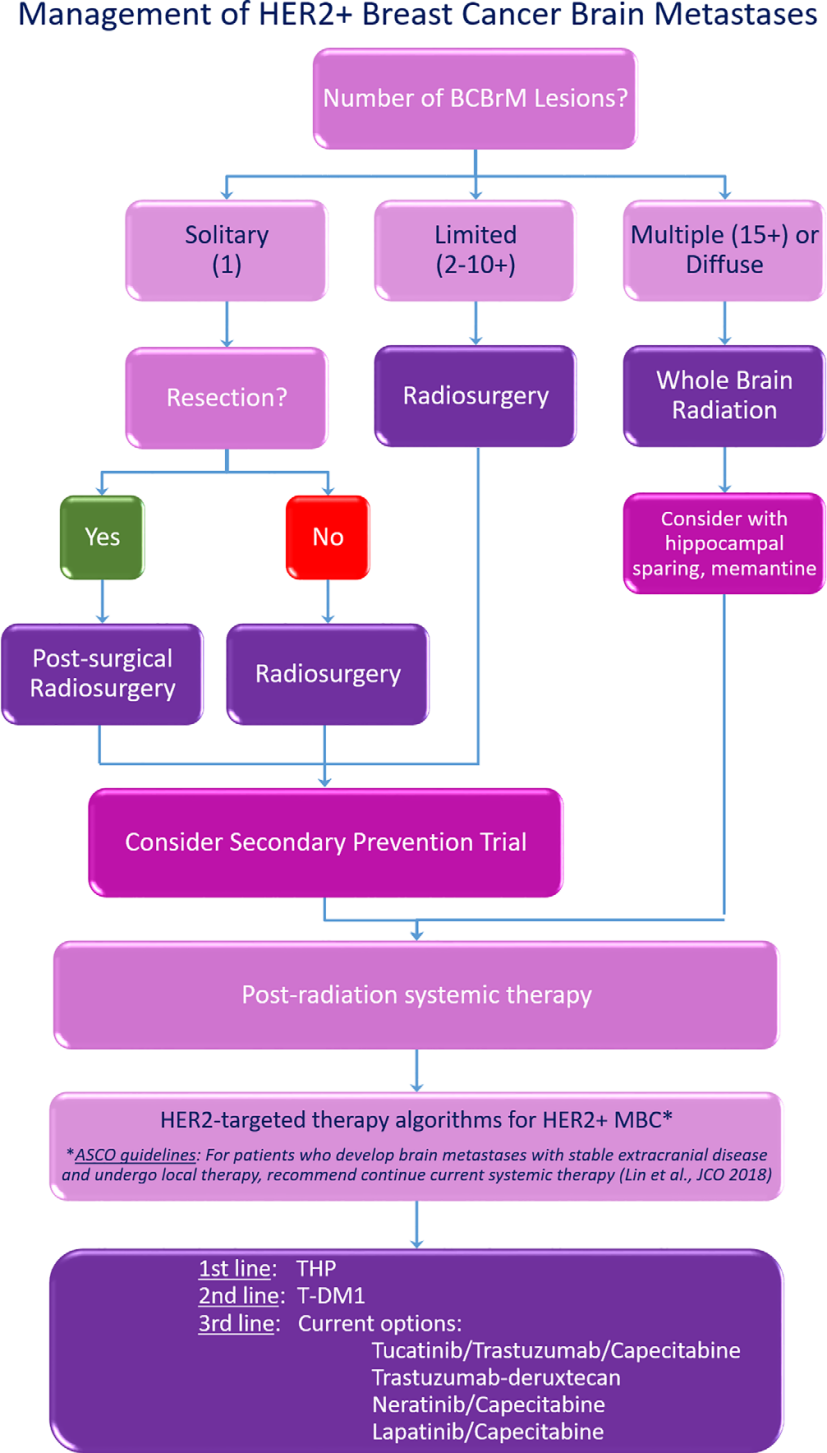
Suggested algorithm for multidisciplinary management of care for patients with HER2+ breast cancer brain metastases. BCBrM: breast cancer brain metastases; MBC: metastatic breast cancer; THP: Taxotere (Docetaxel) + Herceptin (Trastuzumab) + Perjeta (Pertuzumab); T‐DM1: ado‐trastuzumab emtansine (Kadcyla)

The care of patients with HER2+ BCBrM is complex and requires a multidisciplinary team to determine optimal therapy (ie, local or systemic), timing of therapy, and the best management of sequelae of therapy (ie, radiation therapy necrosis) (see algorithm, Figure 1). In addition, as patient symptom burden can be high, incorporation of palliative care early is also recommended as part of the multidisciplinary team, in addition to radiation oncology, medical oncology, and neurosurgery.^73^

### 3.1 Local therapy

Local therapy modalities, including neurosurgical resection, stereotactic radiosurgery (SRS), and/or whole‐brain radiotherapy (WBRT) remain the cornerstone of therapy for BrM.^74^ Neurosurgical resection offers survival benefit when associated with adjuvant radiation therapy, more so in patients with good performance status, controlled systemic disease, and a solitary brain lesion.^75^, ^76^, ^77^ While SRS is preferred in cases with a limited number of BrM, the upper limit of number of lesions remains controversial.^78^ Finally, for patients with multiple, diffuse BrM, WBRT is the recommended treatment modality, but has fallen out of favor in recent years due to observed negative impacts on longer‐term neurocognition.

It is important to highlight that most prospective trials evaluating local treatment of BrM included mainly NSCLC patients, with only a small proportion (~10%‐15%) incorporating breast cancer patients.^79^, ^80^, ^81^ Overall, these trials demonstrated that adding WBRT to initial surgery or SRS decreased intracranial disease recurrence without affecting OS (median 7 to 10 months, *P* = .42 to *P* = .93). Conversely, WBRT has been reported to worsen quality of life and neurocognitive function, particularly in patients with prolonged survival.^82^, ^83^, ^84^ In those cases, neurocognitive decline is progressive and untreatable. Preventive strategies using memantine and hippocampal avoidance have shown improvements in neurocognitive decline.^85^, ^86^

Retrospective analysis of breast cancer patients treated with local therapy for BrM showed that the subtype affects patterns of failure of BrM after treatment with SRS. Luminal HER2+ (HER2+, HR+) patients had a rate of 36% to 38% distant brain new lesions at 1 year, with a median 18 to 22 months OS, while HER2+ cases (HER2+, HR‐) had a rate of 47% to 53% and OS of 11 to 15.4 months.^22^, ^87^ This can help guide discussions clinically, and also begs for systemic therapy agents in the secondary prevention setting to protect against distant brain recurrence after focused radiotherapy in patients with HER2+ BCBrM.

### 3.2 Systemic therapy overview

Systemic therapy for BrM, overall, has shown less efficacy than in systemic, non‐CNS locations. Multiple clinical trials have documented few or no responses using agents with known activity in the metastatic setting.^3^, ^88^, ^89^, ^90^, ^91^, ^92^ In HER2+ BC patients, HER2 targeted agents beyond trastuzumab have been evaluated for their potential therapeutic effect in BrM.

#### 3.2.1 Lapatinib/capecitabine

Lapatinib is a small molecule tyrosine‐kinase inhibitor (TKI) of EGFR and HER2, and is able to cross the BTB.^45^, ^49^ As such, it was the first therapy with promising activity in HER2+ BCBrM patients. However, analysis of lapatinib and capecitabine levels in BrM from patients dosed preoperatively for medically needed craniotomies showed concentrations were very heterogeneous.^93^ When given as a single agent, lapatinib leads to few responses (2%‐6%) and only a small, nonsignificant decrease in the size of BrM lesions.^9^, ^94^ Phase II trials evaluating the combination of lapatinib plus capecitabine in patients with BrM previously treated with WBRT showed an objective response rate of 30%.^9^, ^95^ When the combination was given as first line therapy to 45 patients with low‐volume BrM in the LANDSCAPE trial (single arm phase II), an objective CNS responses of 65.9% (measured by volumetric reduction), median time to CNS progression of 5.5 months, and median time to WBRT of 8.5 months were shown.^96^ However, this treatment was associated with a 49% grade 3 to 4 toxicity rate, mainly represented by diarrhea, hand‐foot syndrome and fatigue. The MA.31 phase II trial randomized 652 patients with HER2+ BC to treatment with either lapatinib plus paclitaxel or trastuzumab plus paclitaxel as first line treatment of metastatic disease.^97^ The trastuzumab combination was superior to the lapatinib combination with median progression free survival (PFS) of 9.0 months and 11.3 months, respectively (HR 1.37, *P* = .001). The incidence of BrM as the first site of progression was 28% for trastuzumab and 20% for lapatinib, with no difference in time to progression between the arms. Considering a potential effect in preventing the development of BrM, a phase III trial of lapatinib plus capecitabine vs capecitabine alone in patients with HER2+ advanced BC who were previously treated with an anthracycline, taxane, and trastuzumab was developed. Patients with baseline BrM were excluded, and only four (2%) patients developed symptomatic BrM as an initial site of progression in the combination therapy arm compared to 13 (6%) patients in the monotherapy group (*P* = .045).^98^

#### 3.2.2 Neratinib/capecitabine

Neratinib is a pan‐HER TKI that targets and inhibits EGFR/HER1, HER2, and HER4 in an irreversible way. Based on the phase II TBCRC 022 trial results, neratinib received an orphan drug designation for HER2+ BCBrM by the FDA in 2019.^99^ In this trial, the combination of neratinib plus capecitabine was evaluated in two cohorts, depending on the previous use of lapatinib. Volumetric BrM response was the primary objective of this clinical trial. In the lapatinib‐naïve cohort (n = 37), 18 (49%) patients had partial responses (PR) and 7 (19%) patients had stable disease (SD) for ≥6 cycles (4.2 months), with median PFS 5.5 months and OS 13.3 months. When RANO‐BM was applied to evaluate responses, 9 (24%) patients had a PR in that cohort. In the cohort of patients that had received lapatinib in the past (n = 12), which was closed for slow accrual, 4 (33%) patients had a PR and 3 (25%) patients had SD for ≥6 cycles (4.2 months), with a PFS of 3.1 months and OS of 15.5 months. Per RANO‐BM evaluation, 2 (17%) patients had a PR. Notable toxicity was observed in both groups, with grade 2 and 3 toxicity levels reported: 62% diarrhea (despite prophylaxis therapy), 24% nausea, 20% vomiting, and 26% fatigue. Overall, neratinib demonstrated a modest effect in the CNS, mostly short‐lived, with non‐negligible toxicity.

#### 3.2.3 Tucatinib/trastuzumab/capecitabine

Tucatinib is a TKI that inhibits HER2 in a reversible way. It has shown promising activity in combination with capecitabine and trastuzumab in a phase I trial, which included notable response in BrM.^100^ Building on that, the HER2CLIMB phase III trial was developed and results were recently reported.^101^ The trial randomized 612 patients with HER2+ metastatic BC previously treated with trastuzumab, pertuzumab and T‐DM1, to trastuzumab and capecitabine, plus tucatinib or placebo. Patients with BrM were allowed to enroll either without or after local therapy, if indicated for symptom control. Patients with progressive BrM and even untreated, asymptomatic BrM were included. The addition of tucatinib to capecitabine and trastuzumab improved PFS at 1 year (33.1% vs 12.3%, HR 0.54; 95% confidence interval [CI], 0.42 to 0.71; *P* < .001), with corresponding improvements in PFS of 7.8 months and 5.6 months, respectively, for the global patient population. OS at 2 years was improved (44.9% vs 26.6%, HR 0.66; 95% CI, 0.50 to 0.88; *P* = .005) with tucatinib, with median OS of 21.9 months and 17.4 months, respectively.

Specific to patients with BrM, 1‐year PFS was 24.9% with addition of tucatinib and 0% in the placebo‐combination group (HR 0.48; 95% CI, 0.34 to 0.69; *P* < .001). Median PFS for those with BrM was 7.6 months with tucatinib vs 5.4 months without tucatinib. The exploratory analysis of intracranial outcomes was also reported recently.^102^ A total of 291 patients with BrM were enrolled in the HER2CLIMB trial, 174 with active BrM, either treated and progressing or untreated, and 117 with treated and stable BrM. The CNS objective response rate in the evaluable subset (55 patients in the tucatinib group and 20 patients in the placebo group) was 47% vs 20%, *P* = .03, with a median duration of response of 6.8 months vs 3.0 months, respectively. The median CNS PFS was also improved by tucatinib, with 9.9 months vs 4.2 months, *P* < .00001, in all BrM patients; with median 9.5 months vs 4.1 months in patients with active BrM, *P* < .0001, and 13.9 months vs 5.6 months in patients with stable BrM, *P* = 0.002. The regimen tucatinib, capecitabine and trastuzumab was approved in April 2020 for adult patients with advanced unresectable or metastatic HER2+ BC, including patients with BrM, who have received one or more prior anti‐HER2‐based regimens in the metastatic setting.

#### 3.2.4 Pertuzumab

Pertuzumab is a MAb that binds to the extracellular domain II of HER2 and inhibits the dimerization of HER2 with other HER family receptors, especially HER3. In this way, it acts in synergy with trastuzumab. It was demonstrated to prolong OS when offered in combination to trastuzumab and docetaxel as first line treatment for metastatic HER2+ BC in the Cleopatra phase III, randomized, controlled trial.^103^ The incidence of BrM as the first site of disease progression was evaluated in an exploratory analysis, and found to be similar in the pertuzumab arm and the placebo arm (13.7% and 12.6%).^104^

Pertuzumab also showed some benefit when added to chemotherapy and trastuzumab in the adjuvant setting in patients with high‐risk HER2+ BC.^105^ In a recent update, with a median 74.1 months follow‐up, the incidence of CNS metastases as invasive disease first recurrence was not different with or without use of adjuvant pertuzumab, 2% in both arms.^106^

#### 3.2.5 TDM‐1 (trasutuzumab emtansine)

T‐DM1 is an ADC containing emtansine (DM1), a microtubule‐inhibitory agent, linked to trastuzumab. It was the first ADC agent to be approved for treatment of HER2+ BC. Several case reports have described the activity of T‐DM1 in CNS metastases.^107^, ^108^ Phase III trials have shown activity of T‐DM1 in HER2+ metastatic disease after previous lines of treatment including trastuzumab and lapatinib, with improvements in both PFS and OS.^109^, ^110^ T‐DM1 is currently the standard therapy for HER2+ BC patients with recurrence or progression of disease after treatment with trastuzumab and pertuzumab. The EMILIA trial randomized advanced HER2+ BC patients, previously treated with trastuzumab and taxanes, to receive either T‐DM1 or the combination of lapatinib plus capecitabine.^110^ Final results showed better PFS (median 9.6 months with T‐DM1 vs 6.4 months with lapatinib plus capecitabine; HR 0.65; 95% CI, 0.55 to 0.77; *P* < .001), and OS (30.9 months vs 25.1 months; HR 0.68; 95% CI, 0.55 to 0.85; *P* < .001) with use of T‐DM1. In a retrospective exploratory analysis of CNS outcomes as part of the EMILIA trial, the rate of CNS progression was similar between the two arms, with CNS metastases as the first site of relapse in 2% of T‐DM1 treated patients and in 0.7% of lapatinib plus capecitabine treated patients, and progression of CNS disease known at baseline in 22.2% and 16%, respectively. Nevertheless, in patients with treated, asymptomatic CNS metastases at baseline, T‐DM1 was associated with improved survival when compared to lapatinib plus capecitabine (median 26.8 vs 12.9 months, HR 0.38 *P* = .008).^111^ This has been interpreted to be due to excellent control of systemic disease, potentially affecting CNS disease progression and OS.

More recently, in the phase III KATHERINE trial, T‐DM1 has been shown to improve invasive disease‐free survival (IDFS) in patients with early HER2+ BC that presented with residual invasive disease after neoadjuvant treatment with trastuzumab and taxanes.^112^ In the interim analysis, with a median follow‐up around 40 months, the rate of distant recurrence was 10.5% with T‐DM1 and 15.9% with trastuzumab. Interestingly, the rate of CNS recurrence as first IDFS event was 5.9% with T‐DM1 and 4.3% with trastuzumab. In a more detailed analysis,^113^ it was found that the incidence of CNS recurrence after first IDFS event was 0.1% with T‐DM1 and 1.1% with trastuzumab, making a total CNS involvement of 6.1% with T‐DM1 and 5.4% with trastuzumab. CNS was the only site of recurrence in 4.8% of T‐DM1 cases and 2.8% of trastuzumab cases. Moveover, the median time to recurrence in the CNS was 17.5 months with T‐DM1 and 11.9 months with trastuzumab. The OS after a CNS event was similar between both treatment groups (12.5 months for T‐DM1 and 14.3 months for trastuzumab, unstratified HR [95% CI] = 1.07 [0.60‐1.91]).^113^ Overall, these findings seem to support the interpretation of the results in the EMILIA CNS subgroup analysis termed competing risk, meaning: “the substantial reduction in the incidence of non‐CNS recurrences as a first event observed with T‐DM1 may be resulting in an increased likelihood of a CNS recurrence as a first event and as the only recurrence.”^113^

#### 3.2.6 Trastuzumab deruxtecan (DS8201)

Trastuzumab deruxtecan, initially known as DS‐8201, is the second ADC to be FDA approved as third line therapy for metastatic HER2+ BC, based on the impressive results of the phase II DESTINY trial.^114^ This ADC is composed of an anti‐HER2 antibody, a cleavable tetrapeptide‐based linker, and a cytotoxic topoisomerase I inhibitor in a 1:8 antibody:cytoxic ratio. In the DESTINY trial, patients with heavily‐pretreated metastatic HER2+ BC with a median of 6 prior lines of therapy all received trastuzumab deruxtecan. The median duration of follow‐up was 11.1 months (range, 0.7 to 19.9). The median response duration to trastuzumab deruxtecan was 14.8 months (95% CI, 13.8 to 16.9), and the median PFS was 16.4 months (95% CI, 12.7 to not reached). Remarkably, PFS for the 24 patients who were enrolled with treated and asymptomatic BrM was 18.1 months (95% CI, 6.7 to 18.1). Adverse events, grade 3 or higher, were most commonly neutropenia (20.7%), anemia (8.7%), and nausea (7.6%). The drug was also associated with interstitial lung disease in 13.6% of the patients (grade 1 or 2, 10.9%; grade 3 or 4, 0.5%; and grade 5, 2.2%), a toxicity which should be monitored and managed aggressively.

## 4 RECOMMENDATIONS

### 4.1 ASCO guidelines in HER2+ BCBM

The American Society of Clinical Oncology (ASCO) has published periodic and timely guidelines for management of BrM in patients with HER2+ advanced BC. Those guidelines are expert consensus‐based recommendations following a targeted, systematic literature review. The most recent guideline was published in 2018 and had similar recommendations to the previous version, published in 2014.^115^ In summary, cases with favorable prognosis and single or limited^2^, ^3^, ^4^ BrM should receive some combination of surgery and radiation, depending on size, resectability and symptoms. When presenting with diffuse BrM, patients should receive WBRT, and if poor clinical prognosis, palliative care is indicated. If progression of CNS disease following initial radiation therapy occurs, some form of local therapy with radiation and/or surgery should be considered when possible, as well as a clinical trial or best supportive care. Systemic therapy should not be switched if systemic extracranial disease is not progressing. Traditional HER2‐targeted therapy algorithms for HER2+ metastatic BC should be offered when systemic disease is progressive.

A suggested algorithm for multidisciplinary management of care for patients with HER2+ BCBrM is presented in Figure 1.

### 4.2 Open questions/next steps

Many questions remain to be answered in the management of HER2+ BCBrM around drug delivery, subtype discordance, and the unique biology of BrM. Obtaining active and homogeneous drug penetration into CNS metastatic lesions is only one obstacle to overcome. To complicate matters, discordance between BC subtype markers have also been demonstrated when BrM are compared to primary tumors.^35^, ^116^ Furthermore, whole‐exome sequencing of patient‐matched BrM and primary tumors demonstrate that metastatic lesions are a product of branched evolution, with mutations “private to the BrM.”^117^ Based on those findings and paving the way of precision medicine strategies into BrM management, a phase II clinical trial proposing genomically‐guided treatment in BrM was developed (NCT03994796). Patients with histologically proven BrM from any solid tumor, including HER2+ BC, are eligible and will be treated based on specific actionable mutations inherent to the BrM. Patients will be matched to brain permeable therapies based on alterations found in their BrM: CDK alterations to abemaciclib, mTOR/AKT/PI3K alterations to the dual mTOR/PI3K inhibitor, paxalisib/GDC‐0084, and NTRK/ROS1 fusions (lung BrM only) to entrectinib. The primary endpoint of this novel clinical trial is overall CNS response rate.

Considering the issues of drug penetration in the CNS and the biologic cascade promoting BrM, preclinical data using a mouse xenograft model of BCBrM demonstrates that temozolomide administered in a preventive fashion can prevent the development of BrM. In these models, temozolomide did not result in reduction in established BrM.^118^ Based on this observation, a phase I/II clinical trial for secondary prevention of BrM in HER2+ BC has been developed, enrolling patients after an initial local therapy to receive T‐DM1 with or without temozolomide, with the goal to prevent and decrease incidence of new BrM.^119^ This represents a new study design of “secondary prevention” in BrM clinical trials which could also be utilized in the development of clinical protocols for other subtypes of BCBrM.

As immunotherapy has emerged as one of the most promising ways to approach cancer therapy over the last decade, HER2+ and TNBC are known to be the most immunogenic subtypes of BC. Biomarkers correlated with immunogenicity such as tumor‐infiltrating lymphocytes (TIL) levels, PD‐L1 expression and tumor mutational burden (TMB) are reportedly more frequent in the HER2+ relative to the luminal subtypes of BC.^120^, ^121^, ^122^ This observation has led to the development of immunotherapy strategies and clinical trials of immunotherapy for advanced HER2+ BC. Though preliminary clinical evidence has shown modest activity of immune checkpoint inhibitors in metastatic HER2+ BC, new strategies and combinations targeting BrM are under evaluation (Table 2). One interesting approach is the use of chimeric antigen receptor‐engineered T cells (CART) to target HER2+ BCBrM, demonstrated to be effective with intraventricular delivery of HER2‐CAR constructs in xenograft models^68^ and currently being investigated in clinical trials (Table 2).

**TABLE 2:** Available clinical trials for HER2‐positive breast cancer brain metastases (BCBrM)

NCT	Title	Intervention	Eligibility
03994796 (Phase II)	Genomically‐guided treatment trial in brain metastases	Palbociclib or GDC‐0084 or entrectinib, dependent on presence of gene mutation	Clinically actionable alteration in NTRK, ROS1, or CDK or PI3K pathway^a^ At least one prior HER2 directed therapy in the metastatic setting
03190967 (Phase I/II)	T‐DM1 alone vs T‐DM1 and metronomic temozolomide in secondary prevention of HER2‐positive breast cancer brain metastases following stereotactic radiosurgery	Phase I: T‐DM1^b^ + temozolomide Phase II: randomization T‐DM1 + or ‐ temozolomide	Phase I: Any number of brain metastases treated with SRS/WBRT within 12 weeks of study entry Phase II: Up to 10 brain metastases treated within 12 weeks of study entry with SRS and/or resection
03417544 (Phase II)	A Phase II study of atezolizumab in combination with pertuzumab plus high‐dose trastuzumab for the treatment of central nervous system metastases in patients with HER2‐positive breast cancer	Trastuzumab + pertuzumab + atezolizumab	At least one measurable CNS metastasis, defined as ≥10 mm in at least one dimension Untreated CNS lesions in asymptomatic patients Treated SRS or surgery with untreated and measurable residual areas Prior WBRT and/or SRS with lesions subsequently progressed are also eligible
03696030 (Phase I)	A Phase 1 cellular immunotherapy study of intraventricularly administered autologous HER2‐targeted chimeric antigen receptor (HER2‐CAR) T cells in patients with brain and/or leptomeningeal metastases from HER2‐positive cancers	HER2‐CAR T^c^ cells via intraventricular administration	Recurrent brain metastases after radiation therapy Recurrent leptomeningeal metastases after intrathecal chemotherapy Untreated brain or leptomeningeal metastases and refuses to undergo radiation and/or intrathecal chemotherapy Eligible to enroll in the study and undergo leukapheresis
02442297 (Phase I)	Phase I Study of intracranial injection of t cells expressing HER2‐specific chimeric antigen receptors (CAR) in subjects with HER2‐positive tumors of the central nervous system (iCAR)	HER2‐CAR T cells via intraventricular administration	HER2‐positive solid tumor metastatic to the CNS
03765983 (Phase II)	Phase II trial of GDC‐0084 in combination with trastuzumab for patients with HER2‐positive breast cancer brain metastases	Trastuzumab + GDC‐0084 (PI3K inhibitor)	Cohort A: At least one measurable CNS metastasis, defined as ≥10 mm in at least one dimension Untreated CNS lesions in asymptomatic patients Treated SRS or surgery with untreated and measurable residual areas prior WBRT and/or SRS with lesions subsequently progressed are also eligible Cohort B: New and/or progressive brain metastasis(es) with clinical indication for resection
01494662 (Phase II)	A Phase II trial of HKI‐272 (neratinib), neratinib and capecitabine, and ado‐trastuzumab emtansine for patients with human epidermal growth factor receptor 2 (HER2)‐positive breast cancer and brain metastases	Different cohorts receiving: neratinib alone, neratinib + capecitabine, neratinib + T‐DM1	Cohort dependent, either resectable brain metastases or not
03933982 (Phase II)	Pyrotinib plus vinorelbine in patients with brain metastases from HER2‐positive metastatic breast cancer: a prospective, single‐arm, open‐label study	Pyrotinib + vinorelbine	At least one CNS metastases with a longest diameter ≥ 1 cm and Controlled CNS symptoms No previous WBRT
03975647 (Phase III)	Randomized, double‐blind, phase 3 study of tucatinib or placebo in combination with ado‐trastuzumab emtansine (T‐DM1) for subjects with unresectable locally‐advanced or metastatic HER2+ breast cancer (HER2CLIMB‐02)	Tucatinib + T‐DM1 vs placebo + T‐DM1	Brain metastases patients allowed with: untreated brain metastases and no need of immediate local therapy previously treated brain metastases, either stable or progressing in no need of immediate local therapy recently treated brain metastases (21 d post WBRT or 28 d after surgical resection)

^a^ NTRK, neurotrophin receptor tyrosine‐kinase; ROS1, ROS proto‐oncogene 1; CDK, cyclin dependent kinase; PI3K, phosphatidylinositol‐3‐kinase.

^b^ T‐DMI1, ado‐trastuzumab emtansine; SRS, stereotactic radiosurgery; WBRT, whole‐brain radiotherapy.

^c^ CAR, chimeric antigen receptor.

## 5 CONCLUSIONS

BrM are a frequent clinical challenge for patients with advanced HER2+ breast cancer. The continuous development of newer, brain permeable, anti‐HER2 therapeutic options has steadily improved the impact of systemic therapy for patients with metastatic HER2+ breast cancer. In parallel, there has been an increased awareness of BrM as a clinically unmet need for this subtype of breast cancer. The complexity of CNS biology and the unique local microenvironment coupled with the historically limited availability of clinical trials for patients with CNS involvement has contributed to a poorer prognosis for this population in the past. This picture is slowly and steadily changing, in large part due to a paradigm shift resulting in the inclusion of patients with BrM in large, randomized, phase 3 clinical trials such as HER2Climb. Enrollment of patients with BrM in future clinical trials evaluating promising, brain permeable, HER2‐targeted therapies should be at the forefront to maintain this forward momentum, with the goal of continuing to improve our patients’ survival and quality of life in a meaningful way.

## ETHICAL STATEMENT

Not applicable.

## AUTHOR CONTRIBUTIONS

**Alexandra Zimmer:** Conceptualization; data curation; project administration; resources; writing‐original draft; writing‐review and editing. **Amanda Van Swearingen:** Data curation; project administration; visualization; writing‐original draft; writing‐review and editing. **Carey Anders:** Conceptualization; data curation; investigation; project administration; resources; supervision; writing‐original draft; writing‐review and editing.

## CONFLICT OF INTEREST

C. K. A.: Research funding PUMA, Lilly, Merck, Seattle Genetics, Nektar, Tesaro, G1‐Therapeutics; Compensated consultant role: Genentech, Eisai, IPSEN, Seattle Genetics; Astra Zeneca; Royalties: UpToDate, Jones and Bartlett.

The other authors have no conflicts requiring disclosure.

## Data Availability

Data sharing is not applicable to this article as no new data were created or analyzed in this study.
